# Recurrence After Liver Resection of Colorectal Liver Metastases: Repeat Resection or Ablation Followed by Hepatic Arterial Infusion Pump Chemotherapy

**DOI:** 10.1245/s10434-020-08776-0

**Published:** 2020-07-09

**Authors:** Florian E. Buisman, Wills F. Filipe, Nancy E. Kemeny, Raja R. Narayan, Rami M. Srouji, Vinod P. Balachandran, Thomas Boerner, Jeffrey A. Drebin, William R. Jarnagin, T. Peter Kingham, Alice C. Wei, Dirk J. Grünhagen, Cornelis Verhoef, Bas Groot Koerkamp, Michael I. D’Angelica

**Affiliations:** 1 Department of Surgery, Erasmus MC Cancer Institute, Erasmus University, Rotterdam, The Netherlands; 2 Department of Medicine, Memorial Sloan Kettering Cancer Center, New York City, NY USA; 3 Department of Surgery, Memorial Sloan Kettering Cancer Center, New York City, NY USA; 4 Department of Surgery, Stanford University, Stanford, CA USA; 5 Hepatopancreatobiliary Service, Department of Surgery, Memorial Sloan Kettering Cancer Center, New York, NY USA

## Abstract

**Background:**

The aim of this study was to investigate the effectiveness of adjuvant hepatic arterial infusion pump (HAIP) chemotherapy after complete resection or ablation of *recurrent* colorectal liver metastases (CRLM).

**Methods:**

A retrospective cohort study was conducted of patients from two centers who were treated with resection and/or ablation of recurrent CRLM only between 1992 and 2018. Overall survival (OS) and hepatic disease-free survival (hDFS) were estimated using the Kaplan–Meier method. The Cox regression method was used to calculate hazard ratios (HRs) with corresponding 95% confidence intervals (CI).

**Results:**

Of 374 eligible patients, 81 (22%) were treated with adjuvant HAIP chemotherapy. The median follow-up for survivors was 65 months (IQR 32–118 months). Patients receiving adjuvant HAIP were more likely to have multifocal disease and receive perioperative systemic chemotherapy at time of resection for recurrence. A median hDFS of 46 months (95% CI 29–81 months) was found in patients treated with adjuvant HAIP compared with 18 months (95% CI 15–26 months) in patients treated with resection and/or ablation alone (*p* = 0.001). The median OS and 5-year OS were 89 months (95% CI 52–126 months) and 66%, respectively, in patients treated with adjuvant HAIP compared with 57 months (95% CI 47–67 months) and 47%, respectively, in patients treated with resection and/or ablation only (*p* = 0.002). Adjuvant HAIP was associated with superior hDFS (adjusted HR 0.599, 95% CI 0.38–0.93, *p* = 0.02) and OS (adjusted HR 0.59, 95% CI 0.38–0.92, *p* = 0.02) in multivariable analysis.

**Conclusion:**

Adjuvant HAIP chemotherapy after resection and/or ablation of recurrent CRLM is associated with superior hDFS and OS.

**Electronic supplementary material:**

The online version of this article (10.1245/s10434-020-08776-0) contains supplementary material, which is available to authorized users.

Repeat resection of colorectal liver metastases (CRLM) is safe and feasible.^1^^–^6 Nearly half of all patients undergo re-resection and/or ablation for intrahepatic recurrences after initial resection of CRLM.^2^^,^^7^ Previous studies have demonstrated favorable overall survival (OS) for highly selected patients after repeat hepatectomy, with a 5-year OS of almost 50%.^8^ Unfortunately, over 60% of patients recur again, involving the liver in 65% of all patients.^6^^,^^9^ Most of these repeat recurrences occur within 2 years after re-intervention.^8^ Effective perioperative systemic or locoregional treatments to reduce or avoid liver recurrence are needed, especially in patients who have already developed liver-only recurrence.

Adjuvant hepatic arterial infusion pump (HAIP) chemotherapy improved hepatic disease-free survival (hDFS) 2 years after CRLM resection in a phase III trial from 60 to 90%.^10^^,^^11^ HAIP chemotherapy involves intra-arterial chemotherapy with floxuridine using a surgically implanted subcutaneous pump. The high first-pass effect of floxuridine allows for a regionally confined high dose of chemotherapy to the liver. The rationale of adjuvant HAIP chemotherapy is that residual micrometastases in the liver after resection can be eliminated with this regional therapy.

The aim of this study was to investigate the outcomes following adjuvant HAIP chemotherapy after resection and/or ablation of recurrent CRLM in the absence of extrahepatic disease.

## Methods

### Patients

Consecutive patients treated between January 1992 and December 2018 at Memorial Sloan Kettering Cancer Center (MSKCC) or between January 2000 and December 2016 at the Erasmus MC Cancer Institute (Erasmus MC) were identified from prospectively maintained liver resection databases. Only patients with recurrent liver-only disease after prior liver resection or ablation were considered for inclusion.

Patients with incomplete resection of the primary or liver tumors were excluded, as were patients with extrahepatic disease present prior to or at the time or hepatic recurrence. Patients treated with HAIP chemotherapy at any other stage than adjuvant for recurrent CRLM were excluded. Patients treated with stereotactic body radiation therapy were also excluded.

Patients were discussed at a multidisciplinary meeting where resection, percutaneous ablation, and open ablation were considered to be curative-intent treatment options. Ablation included both radiofrequency and microwave ablation.

HAIP chemotherapy with floxuridine and concurrent systemic chemotherapy was administered in a similar way to that used after initial resection of CRLM.^12^ A maximum of 6 cycles of adjuvant HAIP chemotherapy was administered, starting 4 weeks after surgery. Perioperative systemic chemotherapy was defined as any chemotherapy received within 6 months prior to or after CRLM resection. Systemic chemotherapy was offered prior to resection in patients with borderline or upfront unresectable CRLM at both centers. At MSKCC, patients with upfront resectable CRLM also received preoperative and/or adjuvant systemic chemotherapy. At Erasmus MC, only patients with early recurrence (within 6 months of primary tumor resection) typically received neoadjuvant systemic chemotherapy. A comparative survival analysis was performed to identify any differences between patients treated with perioperative systemic chemotherapy in both centers.

### Definitions

Clinicopathological data were retrieved from two prospectively maintained databases. Primary tumors were classified as right-sided if arising proximal to the splenic flexure and left-sided if arising at or distal to the splenic flexure. Primary tumors arising at the rectosigmoid junction or distally were considered rectal tumors. The total number of CRLM was determined by the total number of lesions present in the resected specimen as well the total number of lesions ablated. The size of the largest tumor was similarly derived from the pathology report. The disease-free interval was calculated from the time of primary tumor resection to detection of the index CRLM. The recurrence-free interval was defined as the time of resection of the index CRLM to time of detection of the recurrent CRLM. The clinical risk score (CRS) was calculated at initial presentation and used to stratify patients into low risk (CRS 0–2) and high risk (CRS 3–5) of recurrence of disease.^13^ The CRS is the sum of five poor prognostic factors: node-positive primary colorectal tumor, disease-free interval below 12 months, multifocal CRLM, largest tumor greater than 5 cm, and serum carcinoembryonic antigen (CEA) level above 200 µg/l.^13^

### Follow-up

During follow-up at MSKCC after initial hepatectomy, serum CEA measurements and radiological imaging (abdominal and thoracic) were performed every 3–6 months for the first 3 years, and yearly thereafter. At Erasmus MC, follow-up was similar with radiological imaging every 3–6 months for the first 2 years, and yearly thereafter until 5 years.

### Statistical Analysis

Overall survival (OS) was defined as the time from curative treatment of liver recurrence to the time of death or last follow-up, and hDFS was defined from the time of resection and/or ablation of liver recurrence to the time of subsequent liver recurrence, death, or last follow-up. Continuous variables were expressed as medians with interquartile range (IQR) and compared among groups using the Mann–Whitney *U* test. Categorical variables were expressed as proportions and compared among groups using the Chi square test. Kaplan–Meier methods were used to estimate survival, and the log-rank test was used to compare survival across groups. Univariable and multivariable Cox regression analyses were performed to identify factors associated with survival. The total CRS, rather than the individual factors of the CRS, was used in the Cox regression analyses due to the limited number of events per predictor variable. Factors with a *p* value of 0.20 and less were included in the multivariable model. Backward selection with stepwise elimination of factors with a *p* value of more than 0.20 was performed in multivariable Cox regression analyses. A *p* value less than 0.05 was considered statistically significant. Analyses were performed using SPSS (IBM Corp, version 24, Armonk, NY) and RStudio (RStudio, version 1.0.153, Boston, MA). The present study was approved by Institutional Review Boards from both centers.

## Results

### Patients

**Fig. 1:**
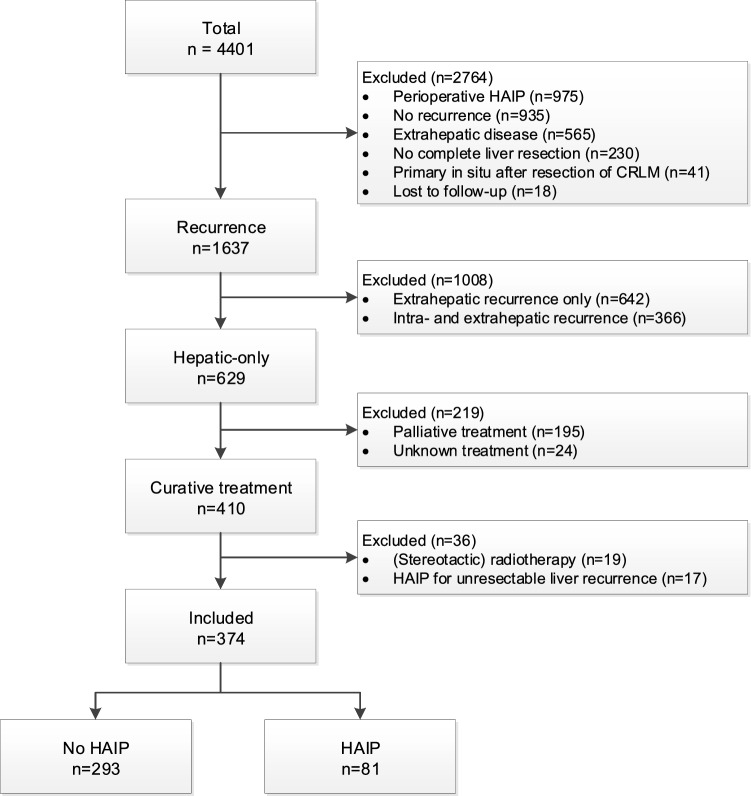
Study flowchart

During the study periods, 3299 patients underwent a curative-intent treatment of CRLM at Memorial Sloan Kettering Cancer Center (MSKCC, New York, United States) and 1102 patients at Erasmus MC Cancer Institute (Erasmus MC, Rotterdam, the Netherlands). A total of 4027 patients were excluded (Fig. 1). The most common reasons for exclusion were perioperative HAIP treatment at time of index CRLM resection (*n* = 975, 22.2%), no recurrence noted in the study period (*n* = 935, 21.1%), extrahepatic recurrence only (*n* = 565, 12.8%), and presence of both intra- and extrahepatic recurrences (*n* = 366, 8.3%). The final group comprised 374 patients, including 81 patients (21.7%) treated with adjuvant HAIP chemotherapy at MSKCC. The majority of patients did not receive HAIP chemotherapy (*n* = 293). These patients were equally distributed between MSKCC (*n* = 148) and Erasmus MC (*n* = 145).

Patient characteristics are summarized in Tables 1 and 2. HAIP patients were younger. More patients treated with HAIP chemotherapy had node positive primary tumors (*n* = 58, 74.4%) compared with no HAIP patients (*n* = 160, 55.9%; *p* = 0.003). The number of recurrent CRLM was higher in HAIP patients (median 2 versus 1, *p* < 0.001). All patients treated with HAIP chemotherapy (*n* = 81, 100%) received perioperative systemic chemotherapy at time of recurrence compared with approximately one-third of patients treated with no HAIP (*n* = 108, 37.5%; *p *< 0.001).

**Table 1:** Baseline characteristics

	All patients	No HAIP	HAIP	*p* value
Total	374	293	81	–
*Patient characteristics*				
Gender				0.005
Male	235	195 (66.6%)	40 (49.4%)	
Female	139	98 (33.4%)	41 (50.6%)	
Center				–
Erasmus MC	143 (38.8%)	143 (49.8%)	–	
MSKCC	231 (61.2%)	150 (51.2%)	81 (100%)	
*Colorectal cancer*				
Primary tumor location				0.24
Right-sided	78 (21.4%)	56 (19.6%)	22 (27.8%)	
Left-sided	175 (48.1%)	138 (48.4%)	37 (46.8%)	
Rectum	111 (30.5%)	91 (31.9%)	20 (25.3%)	
Missing	10			
Pathologic T-stage				0.09
T1–T2	57 (16.4%)	50 (18.1%)	7 (9.7%)	
T3–T4	291 (83.6%)	226 (81.9%)	65 (90.3%)	
Missing	26			
Primary tumor node status				0.003
N0	146 (40.1%)	126 (44.1%)	20 (25.6%)	
N+	218 (59.9%)	160 (55.9%)	58 (74.4%)	
Missing	10			
*Index CRLM*				
Age at resection (median, IQR)	61 (53–69)	63 (56–70)	54 (46–63)	< 0.001
< 70 years	295 (78.9%)	219 (74.7%)	76 (93.8%)	
≥ 70 years	79 (21.1%)	74 (25.3%)	5 (6.2%)	
Disease-free interval				0.14
≤ 12 months	77 (20.6%)	65 (22.3%)	12 (14.8%)	
> 12 months	296 (79.4%) 1	227(77.7%)	69 (85.2%)	
Number of CRLM				0.48
1	150 (41.4%)	120 (42.4%)	30 (38.0%)	
> 1	212 (58.6%)	163 (57.6%)	49 (62.0%)	
Missing	12			
Size of largest CRLM				0.08
≤ 5 cm	296 (88.4%)	230 (86.6%)	66 (94.3%)	
> 5 cm	39 (11.6%)	35 (13.4%)	4 (5.7%)	
Missing	39			
Preoperative CEA				0.61
≤ 200 µg/l	281 (91.2%)	228 (90.8%)	53 (93.0%)	
> 200 µg/l	27 (8.8%)	23 (9.2%)	4 (7.0%)	
Missing	66			
Clinical risk score				0.09
Low risk (0–2)	184 (56.8%)	152 (59.1%)	32 (47.8%)	
High risk (3–5)	140 (43.2%)	105 (40.9%)	35 (52.2%)	
Missing	50			
Positive resection margin				0.15
Yes	46 (12.8%)	38 (13.5%)	7 (9.2%)	
No	294 (81.9%)	231 (82.2%)	62 (81.6%)	
RFA	19 (5.3%)	12 (4.3%)	7 (9.2%)	
Missing	15			
Ablation at time of resection				0.46
Yes	90 (24.1%)	73 (24.9%)	17 (21.0%)	
No	284 (75.9%)	220 (75.1%)	64 (79.0%)	
Perioperative SYS				< 0.001
Yes	277 (77.3%)	203 (69.3%)	74 (92.5%)	
No	96 (25.7%)	90 (30.7%)	6 (7.5%)	
Missing	1			

*CEA* carcinoembryonic antigen, *CRLM* colorectal liver metastases, *Erasmus MC* Erasmus Medical Center, *MSKCC* Memorial Sloan Kettering Cancer Center, *SYS* systemic chemotherapy

**Table 2:** Characteristics at the time of recurrence

Recurrent CRLM	All patients	No HAIP	HAIP	*p* value
Total	374	293	81	–
Recurrence-free interval (median, IQR)	11.0 (7.0–19.3)	11.0 (7.0–20.0)	12.0 (7.0–17.0)	0.91
Number of CRLM (median, IQR)	1 (1–2)	1 (1–2)	2 (1–2)	< 0.001
Missing	16			
Size of largest CRLM (median, IQR)	2.1 (1.5–3.0)	2.1 (1.5–3.1)	2.1 (1.6–2.1)	0.78
Missing	61			
CEA at recurrence (median, IQR)	6.4 (3.0–15.2)	6.9 (3.0–16.4)	6.3 (2.9–13.3)	1.00
Missing	94			
Treatment				< 0.001
Resection only	252 (67.4%)	175 (59.7%)	77 (95.1%)	
Resection with ablation	22 (5.9%)	19 (6.5%)	1 (1.2%)	
Ablation only	100 (26.7%)	99 (33.8%)	3 (3.7%)	
Perioperative SYS				< 0.001
Yes	189 (51.2%)	108 (37.5%)	81 (100%)	
No	180 (48.8%)	180 (62.5%)		
Missing	2			

*CEA* carcinoembryonic antigen, *CRLM* colorectal liver metastases, *IQR* interquartile range, *SYS* systemic chemotherapy

### Survival Outcomes

Median follow-up for survivors was 65 months (95% CI 57–73 months), and 190 patients (50.8%) died during follow-up. Duration of follow-up was similar between HAIP patients (73 months, 95% CI 56–90) and no HAIP patients (62 months, 95% CI 52–72). No differences were found for OS (*p* = 0.65) in patients from either center that were treated with perioperative systemic chemotherapy (Supplementary Figure 1). In addition, no differences were found for OS (*p* = 0.59) in patients that were treated with resection with/or without ablation versus ablation only.

### Hepatic Disease-Free Survival

**Fig. 2:**
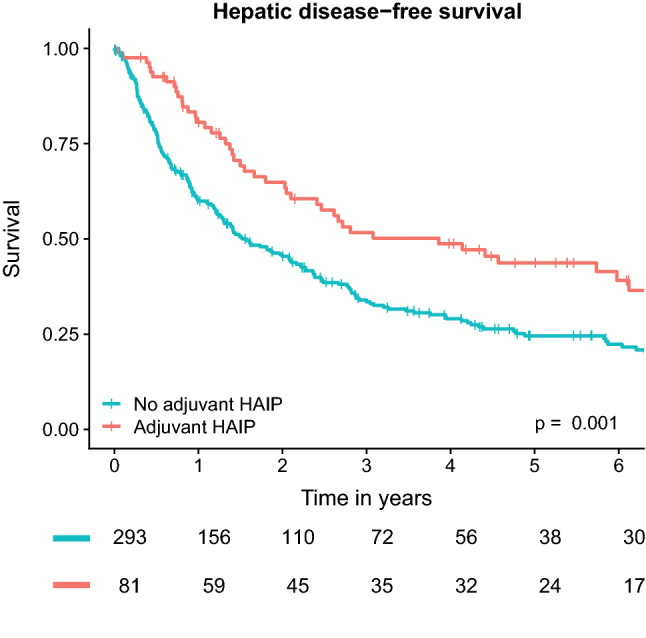
Kaplan-Meier analysis for hepatic disease-free survival

The median hDFS was 46 months (95% CI 29–81 months) for patients treated with HAIP chemotherapy compared with 19 months (95% CI 15–26 months) for patients treated without HAIP chemotherapy (*p* = 0.001, Fig. 2). On univariable analysis, recurrence-free interval (HR 0.99, 95% CI 0.98–1.00, *p* = 0.03), preoperative CEA level at recurrence (HR 1.01, 95% CI 1.00–1.01, *p* = 0.01), ablation only procedures (HR 1.80, 95% CI 1.37–2.37, *p *< 0.001), and HAIP chemotherapy treatment (HR 0.60, 95% CI 0.43–0.82, *p* = 0.001) were associated with hDFS (Supplementary Table 1). On multivariable analysis, the number of CRLM at the time of recurrence (adjusted HR 1.23, 95% CI 1.06–1.42, *p* = 0.006), ablation only procedure (adjusted HR 1.56, 95% CI 1.09–2.24, *p* = 0.02), and HAIP chemotherapy treatment (adjusted HR 0.59, 95% CI 0.38–0.93, *p* = 0.02) were the only independent prognostic factors for hDFS.

### Overall Survival

**Fig. 3:**
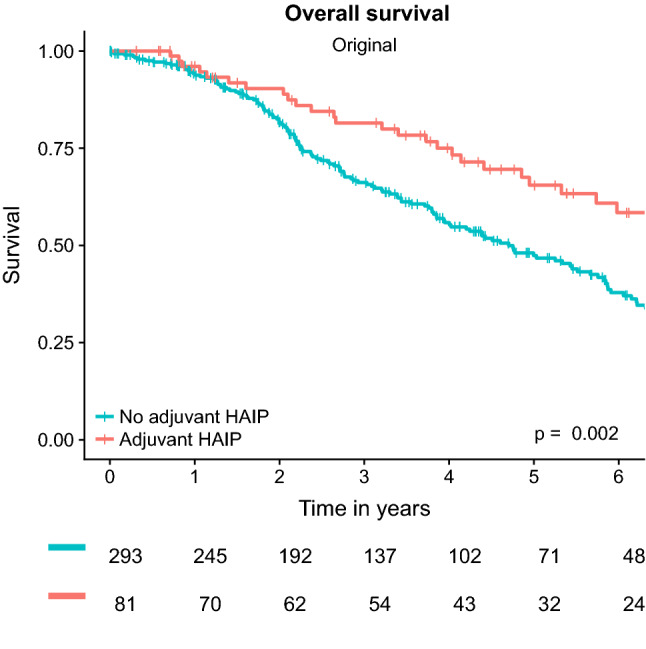
Kaplan-Meier analysis for overall survival

The median OS was 92 months (95% CI 64–120 months) for patients treated with HAIP chemotherapy compared with 57 months (95% CI 47–67 months) for patients treated without HAIP chemotherapy (*p* = 0.002, Fig. 3). The 5-year OS was 66% in HAIP patients compared with 47% in no HAIP patients. Prognostic factors associated with OS on univariable analysis were positive resection margin at the time of index CRLM resection (HR 1.79, 95% CI 1.17–2.27, *p* = 0.007), elevated CEA level at recurrence (HR 1.01, 95% CI 1.00–1.01, *p* < 0.001), and adjuvant HAIP chemotherapy treatment (HR 0.56, 95% CI 0.38–0.82, *p* = 0.003, Table 3). On multivariable analysis, the CEA level at the time of recurrent CRLM detection (adjusted HR 1.01, 95% CI 1.00–1.01, *p* = 0.004) and HAIP chemotherapy treatment (adjusted HR 0.59, 95% CI 0.38–0.92, *p* = 0.02) remained independent prognostic factors for OS.

**Table 3:** Univariable and multivariable Cox regression analysis of factors associated with overall survival

	Univariable			Multivariable		
	HR	95% CI	*p* value	HR	95% CI	*p* value
Index CRLM resection						
Age (> 70 years)	1.27	0.89–1.81	0.19			
Right-sided tumor	0.94	0.64–1.37	0.73			
Pathologic T-stage (T3–T4)	0.97	0.64–1.47	0.89			
Clinical risk score (High)	0.97	0.71–1.34	0.87			
Resection margin (R1)	1.79	1.17–2.27	0.007	1.59	0.97–2.61	0.07
Recurrent CRLM resection						
Recurrence-free interval*	0.99	0.98–1.00	0.11			
Number of recurrent CRLM*	1.07	0.94–1.22	0.29			
Diameter of recurrent CRLM*	1.01	0.91–1.12	0.86			
CEA at recurrence*	1.01	1.00–1.01	< 0.001	1.01	1.00–1.01	0.004
Ablation only procedure	1.26	0.90–1.76	0.18			
Perioperative SYS	1.20	0.89–1.61	0.24			
Adjuvant HAIP	0.56	0.38–0.82	0.003	0.59	0.38–0.92	0.02

*SYS* systemic chemotherapy, *CEA* carcinoembryonic antigen, *CRLM* colorectal liver metastases

*Continuous

## Discussion

This study found that patients receiving adjuvant HAIP chemotherapy after resection and/or ablation of recurrent CRLM had superior hDFS and OS. Patients who received adjuvant HAIP chemotherapy were younger, had more advanced disease, and were more likely to receive perioperative systemic chemotherapy. However, adjuvant HAIP chemotherapy was an independent prognostic factor in multivariable analysis for both hDFS (adjusted HR 0.51, *p* = 0.002) and OS (adjusted HR: 0.59, *p* = 0.02).

In a previous study, we found that perioperative systemic chemotherapy had no impact on the intrahepatic recurrence rate after initial resection of CRLM.^14^ Therefore, it seems unlikely that it would be beneficial in the setting of liver-only recurrence. Adjuvant HAIP chemotherapy has been shown to significantly decrease the hepatic recurrence rate and overall recurrence rate after initial resection of CRLM in randomized controlled trials.^10^^,^^15^ Moreover, adjuvant HAIP was associated with improved median OS from 44 months to 67 months in a retrospective study with 2368 patients.^16^ Outcomes from treatment of recurrent CRLM with adjuvant HAIP chemotherapy have not been studied. The rationale for adjuvant HAIP chemotherapy after resection and/or ablation of recurrences confined to the liver is that these patients have demonstrated a propensity for liver-confined metastatic disease, which may explain the favorable results of HAIP found in our study of these patients.

The safety and effectiveness of repeat hepatectomy in selected patients have been reported in several studies.^1^^–^6 With proper selection, repeat hepatectomy is considered safe, with similar mortality and morbidity to the initial hepatectomy. In well-selected patients, median OS after second hepatectomy has been reported to range from 32 to 43 months,^2^^,^^6^^,^^8^^,^^17^ and 5-year OS rates ranged from 30% to 48%.^3^^,^^6^^,^^8^ A systematic review and meta-analysis of 22 studies, including 1610 patients, found a median OS after hepatectomy for recurrent disease of 35 months and a 5-year OS of 42%.^6^ Notably, the median OS of patients not treated with adjuvant HAIP chemotherapy in our study was 57 months, and the 5-year OS was 47%. This superior survival in our study, compared with historical cohorts, may be attributable to the strict inclusion criteria of our study, excluding patients with prior extrahepatic disease or extrahepatic recurrence at the time of intrahepatic recurrence. Patients with extrahepatic disease were excluded because a previous study found no benefit in OS of HAIP in patients with extrahepatic disease.^16^

Previous studies identified factors associated with worse OS to include CRLM larger than 5 cm at initial hepatectomy, age below 40 years at initial hepatectomy, more than 5 liver tumors at repeat hepatectomy, and major hepatectomy at time of repeat resection.^1^^,^^5^ A concern about previous studies is their small sample size, limiting the power of their analyses. None of these previously identified prognostic factors at the time of initial hepatectomy was associated with OS in multivariable analysis in our study. In addition to the administration of HAIP chemotherapy, we also found that CEA level (adjusted HR 1.01, 95% CI 1.00–1.01, *p* = 0.004) was independently associated with OS. The number of CRLM at the time of recurrence (adjusted HR 1.23, 95% CI 1.06–1.42, *p* = 0.006), ablation only procedures (adjusted HR 1.56, 95% CI 1.09–2.24, *p* = 0.02), and HAIP chemotherapy treatment (adjusted HR 0.59, 95% CI 0.38–0.93, *p* = 0.02) were the only independent prognostic factors for hDFS.

In the current study, patients treated with resection and/or ablation were included. Two small studies compared these approaches in patients with recurrent CRLM.^4^^,^^8^ The first retrospective study evaluated 64 patients and found similar OS in patients treated with resection (*n* = 31, 33 months) or open/percutaneous ablation (*n* = 33, 33 months; *p* = 0.45).^4^ Another retrospective study of 91 patients found similar results with a 5-year OS of 52% in patients treated with resection compared with 53% in patients treated with percutaneous ablation.^8^ A limiting factor is the absence of pathological confirmation of CRLM diagnosis after ablation-only procedures, which comprised one-third (*n* = 99, 33.8%) of patients in the no HAIP group in the current study. More patients in the no HAIP group were treated with ablation only (34% versus 4%) at time of liver recurrence. However, similar OS was found in patients treated with resection (with or without ablation) or ablation only at time of liver recurrence (*p* = 0.59). In addition, no difference was found in the number of ablations in the no HAIP group (*n* = 73, 25%) compared with the HAIP group (*n* = 17, 21%) (*p* = 0.46) at time of initial CRLM treatment. No association of ablative procedures without resection and OS (HR 1.26, 95% CI 0.90–1.76, *p* = 0.18) could be demonstrated.

In the present study, all patients receiving HAIP chemotherapy were concomitantly treated with systemic chemotherapy. Therefore, this study did not evaluate the effectiveness of HAIP chemotherapy alone. Moreover, different regimes were used over time due to the availability of newer chemotherapy regimens relatively recently. Limited evidence is available on the value of perioperative systemic chemotherapy in patients with repeat hepatectomy.^7^ In our study, perioperative systemic chemotherapy was not associated with survival in multivariable analysis (HR 1.20, *p* = 0.24).

A limitation of this study was the extensive period of inclusion. During this period, the selection criteria for re-resection likely changed as well as the available perioperative systemic chemotherapy agents.^2^ However, factors such as number of CRLM, size of CRLM, and CEA level were included in the multivariable analysis, adjusting for this time effect. Moreover, systemic chemotherapy (regardless of the regimen) was not associated with OS. Another limitation of this study was the absence of genomic data (*KRAS* and *BRAF* mutations). These genomic alterations may have influenced survival. However, previous studies have demonstrated that the effect of HAIP chemotherapy is independent of KRAS mutational studies.^18^ Other studies demonstrated that RAS mutations are associated with unsalvageable recurrences after initial hepatectomy; this may also apply for subsequent recurrences after curative treatment of recurrent CRLM.^19^ However, primary tumor location, which is associated with KRAS mutations, and inferior survival in right-sided patients in previous studies, were included in multivariable analysis in this paper.^20^ The use of tumor location likley makes up for the absence of KRAS mutational status in our study. Furthermore, it has also been shown that BRAF rarely presents with isolated and resectable disease, making it unlikely that BRAF would have been a relevant factor for these patients.^21^

In addition, it is unknown whether treatment of subsequent recurrences differed between the two centers. Since all patients treated with adjuvant HAIP chemotherapy for liver recurrence originated from MSKCC, any difference in treatment of subsequent recurrences could have introduced bias. Furthermore, HAIP chemotherapy was administered at the discretion of the treating medical oncologists and surgical oncologists. HAIP chemotherapy requires regular outpatient clinic visits (every 2 weeks) for refill of the pump; this is not possible for most patients living far away from MSKCC. Relative contra-indications for HAIP chemotherapy are patients with a completely replaced right and left hepatic artery, patients with partial portal vein thrombosis, and patients with extrahepatic disease.

This is the first study reporting on the effectiveness of adjuvant HAIP chemotherapy in patients after resection and/or ablation of recurrent CRLM. Our findings suggest that a prospective trial is indicated to investigate the favorable hDFS and OS of adjuvant HAIP after resection and/or ablation of recurrent CRLM.

In conclusion, this retrospective study found that HAIP is independently associated with superior hDFS and OS after resection or ablation for isolated recurrent CRLM.

## Electronic supplementary material

Below is the link to the electronic supplementary material.

Supplementary material 1 (DOCX 28 kb)

Supplements Figure 1. Kaplan–Meier analysis for overall survival in patients treated with perioperative systemic chemotherapy stratified by center (EPS 715 kb)
