# Supplementary material for: Recurrence After Liver Resection of Colorectal Liver Metastases: Repeat Resection or Ablation Followed by Hepatic Arterial Infusion Pump Chemotherapy
Source: Ann Surg Oncol. 2020 Jul 9;28(2):808–16. doi: 10.1245/s10434-020-08776-0 (PMC7801355; doi:10.1245/s10434-020-08776-0)
Supplement: Supplementary file 1 — Supplementary material 1 (DOCX 28 kb) [file 10434_2020_8776_MOESM1_ESM.docx]

**Supplements**

**Supplements Table 1. Univariable and multivariable Cox regression analysis of factors associated with hepatic disease-free survival**

|  | **Univariable** | | | **Multivariable** | | |
| --- | --- | --- | --- | --- | --- | --- |
|  | **HR** | **95% CI** | **P-value** | **HR** | **95% CI** | **P-value** |
| *Index CRLM resection* |  |  |  |  |  |  |
| Age (>70 years) | 1.24 | 0.93-1.66 | 0.15 |  |  |  |
| Right-sided tumor | 1.14 | 0.82-1.59 | 0.44 |  |  |  |
| Pathologic T-stage (T3-T4) | 0.99 | 0.70-1.41 | 0.97 |  |  |  |
| Clinical risk score (High) | 1.28 | 0.98-1.67 | 0.08 | 1.30 | 0.94-1.79 | 0.11 |
| Resection margin (R1) | 1.38 | 0.96-1.99 | 0.09 |  |  |  |
| *Recurrent CRLM resection* |  |  |  |  |  |  |
| Recurrence-free interval* | 0.99 | 0.98-1.00 | 0.03 |  |  |  |
| Number of recurrent CRLM* | 1.10 | 0.98-1.22 | 0.12 | 1.23 | 1.06-1.42 | 0.006 |
| Diameter of recurrent CRLM* | 1.00 | 0.92-1.10 | 0.92 |  |  |  |
| CEA at recurrence* | 1.01 | 1.00-1.01 | 0.01 |  |  |  |
| Ablation only procedure | 1.80 | 1.37-2.37 | <0.001 | 1.56 | 1.09-2.24 | 0.02 |
| Perioperative SYS | 0.96 | 0.75-1.23 | 0.75 |  |  |  |
| Adjuvant HAIP | 0.60 | 0.43-0.82 | 0.001 | 0.59 | 0.38-0.93 | 0.02 |

Abbreviations: SYS: systemic chemotherapy, CEA: carcinoembryonic antigen, CRLM: colorectal liver metastases

*Continuous
